# Improved surface passivation and reduced parasitic absorption in PEDOT:PSS/c-Si heterojunction solar cells through the admixture of sorbitol

**DOI:** 10.1038/s41598-019-46280-y

**Published:** 2019-07-05

**Authors:** Marc-Uwe Halbich, Dimitri Zielke, Ralf Gogolin, Rüdiger Sauer-Stieglitz, Wilfried Lövenich, Jan Schmidt

**Affiliations:** 1 Institute for Solar Energy Research Hamelin (ISFH), Am Ohrberg 1, 31860 Emmerthal, Germany; 2 Heraeus Deutschland GmbH&CoKG, Electronic Chemicals, Chempark Leverkusen, 51368 Leverkusen, Germany; 3 Department of Solar Energy, Institute of Solid-State Physics, Leibniz University Hannover, Appelstr. 2, 30167 Hannover, Germany

**Keywords:** Electronic devices, Solar cells

## Abstract

We examine the impact of sorbitol admixture to the hole-conduction polymer PEDOT:PSS [poly(3,4-ethylenedioxythiophene):poly(styrene sulfonate)] on the characteristics of PEDOT:PSS/crystalline silicon heterojunction solar cells. We fabricate solar cells where the PEDOT:PSS layer is deposited as a hole-collecting contact at the cell rear, whereas the electron-collecting front is conventionally processed by means of phosphorus diffusion. Surprisingly, we observe that the admixture of the infrared-transparent sorbitol not only improves the short-circuit density of the solar cells due to the reduction of the infrared parasitic absorption, but also improves the passivation quality of PEDOT:PSS on silicon and hence the open-circuit voltage of the solar cells. The series resistance is not influenced by the admixture of sorbitol up to 4.0 wt.% sorbitol admixture in the PEDOT:PSS dispersion, but shows a pronounced increase for larger sorbitol contents. The optimal sorbitol content concerning efficiency is hence 4.0 wt.%, leading to an energy conversion efficiency of 20.4% at one sun, which is more than 1% absolute higher compared to the efficiency of the reference cells without sorbitol.

## Introduction

Heterojunction solar cells combining the hole-conducting polymer poly(3,4-ethylenedioxythiophene):poly(styrene sulfonate) [PEDOT:PSS] and c-Si have demonstrated their high-efficiency potential in previous studies^1–9^. In the first fabricated PEDOT:PSS/c-Si solar cells, the organic layer was deposited to the front, leading to relatively low short-circuit current densities *J*_sc_ due to the parasitic absorption losses within the PEDOT:PSS. Moving the organic layer to the rear in the so-called ‘BackPEDOT’ cell concept^10^ resulted in significantly improved *J*_sc_ and led for the first time to efficiencies exceeding 20%^11,12^. Still it turned out that even for the BackPEDOT cell, *J*_sc_ is limited by parasitic free-carrier absorption within the PEDOT:PSS layer of infrared photons reaching the cell rear^10^. In the present study, we examine a promising and easy-to-implement approach to reduce the parasitic absorption in BackPEDOT cells by increasing the transparency of the PEDOT:PSS layer by adding sorbitol to the precursor dispersion. In a previous study^13^, we showed that sorbitol significantly increases the transparency of the PEDOT:PSS layer in the infrared and thus sorbitol can be used for effectively reducing the parasitic absorption in the PEDOT:PSS layer. Sorbitol is already established as a conductivity additive in PEDOT:PSS^14,15^. In this work, we fabricate solar cells to examine the impact of the sorbitol admixture on device level. In addition, we also examine the impact on surface passivation on lifetime samples. Our experimental results clearly show that the sorbitol admixture does not only positively affect the *J*_sc_ of the cells, but due to an improved surface passivation, shows also a positive impact on the open-circuit voltage *V*_oc_. Hence, the addition of sorbitol to the PEDOT:PSS dispersion has multiple advantages for PEDOT:PSS/c-Si heterojunction cells.

## Experimental Details

### Lifetime sample preparation

In order to assess the passivation quality of the PEDOT:PSS/c-Si interface, contactless lifetime samples are fabricated on 300 µm thick (100)-oriented *p*-type float-zone silicon (FZ-Si) wafers with a resistivity of 200 Ωcm. After RCA cleaning, one wafer surface is passivated by a 100 nm thick plasma-enhanced-chemical-vapor-deposited (PECVD) SiN*x* layer (Plasmalab 80 Plus, Oxford) with a refractive index of 2.4 at a deposition temperature of 400 °C. After SiN_*x*_ deposition, the samples are dipped in 1% hydrofluoric acid (HF) for 60 seconds. The PEDOT:PSS dispersion (Clevios special grade, Clevios Heraeus GmbH), which has a solid content of 2.2 wt.% of PEDOT:PSS in water, was mixed with 1.0 to 18.0 wt.% sorbitol and stirred overnight at room temperature on a magnetic stirrer. Immediately after the HF dip, the PEDOT:PSS dispersion is deposited by spin coating at 500 revolutions per minute (rpm) for 10 seconds and subsequently 1500 rpm for 30 seconds. Subsequently, the PEDOT:PSS layer is annealed in ambient environment at a temperature of 130 °C for 10 min. Injection-dependent measurements of the effective carrier lifetime τ_eff_(Δ*n*) are performed using a Sinton Lifetime Tester (WCT-120, Sinton Instruments). The recombination current density parameter (also sometimes denoted ‘saturation current density’) *J*_0_ of each measured sample is extracted from the slope of the inverse lifetime 1/τ_eff_ vs. the excess carrier density Δ*n* curve^16^.

### Solar cell fabrication

**Figure 1:**
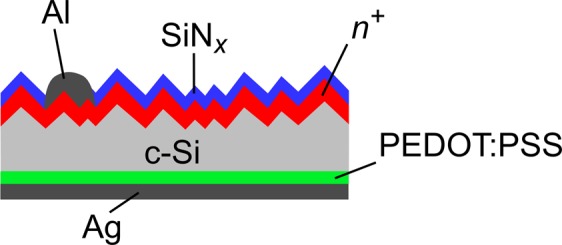
Cross-section of a BackPEDOT solar cell.

Figure 1 shows the cross-section of a BackPEDOT solar cell. For the solar cell fabrication we use 300 µm thick 6″ (100)-oriented *p*-type boron-doped FZ-Si wafers with resistivities of 0.5 and 1.3 Ωcm, respectively. After RCA cleaning and protecting both surfaces by a 200 nm thick dielectric SiO_2_ layer, 2 × 2 cm^2^ diffusion windows are opened by laser ablation on one wafer surface. After one more RCA cleaning sequence, the silicon surface within the ablated window is random-pyramid-textured using a KOH/isopropanol solution. Subsequently, an *n*^+^ emitter with a sheet resistance in the range 92–110 Ω/sq is formed by phosphorus in-diffusion in a quartz-tube furnace (TS 81004, Tempress) at 830 °C in a POCl_3_/O_2_ atmosphere. The wafers are then laser-cut into 2.49 × 2.49 cm^2^ large samples, and after additional cleaning, the phosphorus silicate glass is removed in a 1% HF solution. An 0.24 nm thick AlO_*x*_ tunneling layer is then deposited by means of plasma-assisted atomic layer deposition (FlexAL, Oxford Instruments) on the front surface. Next, an aluminum grid is deposited on the cell front through a nickel shadow mask by electron beam evaporation (BAK 550, Balzers). The front surface is then coated by a surface-passivating PECVD SiN_*x*_ layer (Plasmalab 80 Plus, Oxford) with a refractive index of 2.4 (6 nm) and on top of that by a SiN_*x*_ antireflection coating with a refractive index of 1.9 (75 nm). Subsequently, the cells are annealed for 2 min at 320 °C in order to improve the front surface passivation and for contact formation^17^. Before depositing the PEDOT:PSS to the cell rear, the dielectric SiO_2_ protection layer at the rear is removed using 40% HF. The PEDOT:PSS dispersion (Clevios special grade, Clevios Heraeus GmbH), which has a solid content of 2.2 wt.%, was mixed with 1.0 to 7.7 wt.% sorbitol and stirred overnight on a magnetic stirrer. The PEDOT:PSS dispersion is deposited by spin-coating at 500 rpm for 10 seconds and subsequently 12000 rpm for 30 seconds. The coated PEDOT:PSS layer is annealed in ambient environment at 130 °C for 10 min. Finally, the rear surface is full-area metallized by a 1 µm thick silver layer deposited using electron beam evaporation (BAK 550, Balzers).

## Results and Discussion

### Surface passivation

**Figure 2:**
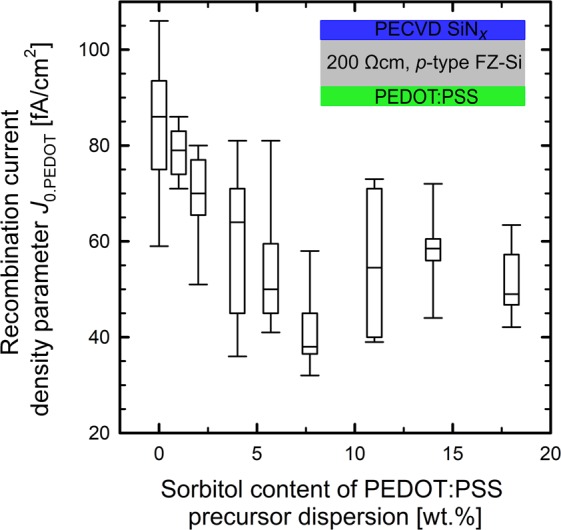
Box plot of the recombination current density parameter *J*_0__.__PEDOT_ of the PEDOT:PSS/c-Si interface as a function of the sorbitol content of the PEDOT:PSS precursor dispersion. PEDOT:PSS was applied to 9 test samples for each sorbitol concentration. The *J*_0__.__SiN_ value of 16 fA/cm^2^ of the SiN_*x*_-passivated silicon surface was subtracted from the measured *J*_0_ value to account only for recombination at the PEDOT:PSS/c-Si interface.

Figure 2 shows a box plot of the measured recombination current density parameter *J*_0.PEDOT_ of the PEDOT:PSS/c-Si interface as a function of the sorbitol content added to the PEDOT:PSS precursor dispersion.

Table 1 shows the PEDOT:PSS layer thicknesses of the lifetime samples shown in Fig. 2. The correponding total sheet resistances are also shown in Table 1. The reduction of the sheet resistance by adding more than 1.0 wt.% sorbitol agrees well with the behaviour reported in the literature that sorbitol serves as a conductivity-increasing additive^14,15^. The PEDOT:PSS layer thicknesses were determined using a profilometer (Dektak 150, Veeco) and the corresponding sheet resistances were measured using a Sinton lifetime tester (WCT-120, Sinton Instruments). *J*_0.PEDOT_ was extracted from the measured overall *J*_0_ values by subtraction of the *J*_0.SiN_ value of the SiN_*x*_-passivated wafer surface. *J*_0.SiN_ was determined on both-sides-SiN_*x*_-passivated wafers with a median value of 16 fA/cm^2^. For silicon surfaces passivated with PEDOT:PSS without admixture of sorbitol, we measure a median *J*_0.PEDOT_ value of 86 fA/cm^2^. We observe that with increasing sorbitol content of the PEDOT:PSS precursor dispersion the measured *J*_0.PEDOT_ decreases. At a sorbitol concentration of 4.0 wt.% in the precursor dispersion, we measure a median *J*_0.PEDOT_ value of 64 fA/cm^2^. At at a sorbitol concentration of 7.7 wt.%, a median *J*_0:PEDOT_ of only 38 fA/cm^2^ is achieved, which means that the recombination at the PEDOT:PSS/c-Si interface is more than halved by adding 7.7 wt.% of sorbitol to the precursor solution. At even higher sorbitol concentrations of 11 wt.% and 14 wt.% the *J*_0.PEDOT_ value rises to a median of 55 fA/cm^2^ and 58 fA/cm^2^ respectively. A sorbitol concentration of 18 wt.% provides a *J*_0.PEDOT_ median of 49 fA/cm^2^. According to our results a saturation in *J*_0.PEDOT_ at high sorbitol concentrations can be observed. Nardes *et al*.^18^ have shown that the addition of sorbitol reduces the work function of PEDOT:PSS for sorbitol concentration of 5 wt.%, and for higher sorbitol concentrations a saturation in the work function occurs. The positive impact of sorbitol on the electronic passivation properties of PEDOT:PSS on c-Si might be due to a change in the electronic band structure of the organic solution. It is well known from the literature that the addition of sorbitol to the PEDOT:PSS precursor dispersion reduces the work function of the PEDOT:PSS with increasing sorbitol content^18–21^. Our results here clearly prove that adding sorbitol to the PEDOT:PSS precursor solution significantly improves the c-Si surface passivation quality.

**Table 1:** Mean PEDOT:PSS layer thicknesses measured using a profilometer (Dektak 150, Veeco) and total sheet resistances of the lifetime samples measured using a Sinton lifetime tester (WCT-120, Sinton Instruments) for PEDOT:PSS applications at spin coating speeds of 1500 and 12000 rpm, respectively.

Sorbitol content in the PEDOT:PSS precursor dispersion [wt.%]	PEDOT:PSS layer thickness [nm]		Total sheet resistance [Ω/□]	
	PEDOT:PSS applied at 1500 rpm	PEDOT:PSS applied at 12000 rpm	PEDOT:PSS applied at 1500 rpm	PEDOT:PSS applied at 12000 rpm
0.0	1674 ± 343	181 ± 60	2269	2518
1.0	978 ± 123	140 ± 36	1851	2056
2.0	1070 ± 125	146 ± 32	124	2083
4.0	1623 ± 240	162 ± 41	67.3	435
5.7	2137 ± 237	163 ± 39	61.5	304
7.7	2087 ± 322	184 ± 55	60.2	274
11.0	1033 ± 47	—	99.2	—
14.0	1766 ± 52	—	76.6	—
18.0	1936 ± 81	—	79.6	—

The PEDOT:PSS layer thickness measurements were performed in 1 mm steps over a length of 2 cm (20 data points) centered in the sample center. The error bars result from the deviation of the mean value.

### Solar cells

A total of 42 PEDOT:PSS/c-Si solar cells with an area of 2 × 2 cm^2^ were fabricated. The precursor dispersion of the spin-coated PEDOT:PSS layer on the solar cell rear was mixed with different contents of sorbitol and stirred overnight on a magnetic stirrer. We examine the impact of the sorbitol admixture on the most relevant solar cell parameters that are the open-circuit voltage *V*_oc_, the short-circuit current density *J*_sc_, the fill factor *FF* and the series resistance *R*_s_ as well as the energy conversion efficiency η. The *J*_sc_ − *V*_oc_ characteristics as well as the illuminated current-voltage (*J*–*V*) curves are measured under standard testing conditions at 1 sun and 25 °C using a LOANA measurement system (pv-tools, Hamelin, Germany). The series resistance *R*_s_ is determined from the measured fill factor *FF*, pseudo-fill factor *pFF* as well as the measured *J*_sc_ and *V*_oc_ values using the equation:

$${\rm{F}}{\rm{F}}={\rm{p}}{\rm{F}}{\rm{F}}(1-\frac{{{\rm{R}}}_{{\rm{s}}}{{\rm{J}}}_{{\rm{s}}{\rm{c}}}}{{{\rm{V}}}_{{\rm{o}}{\rm{c}}}})$$

**Figure 3:**
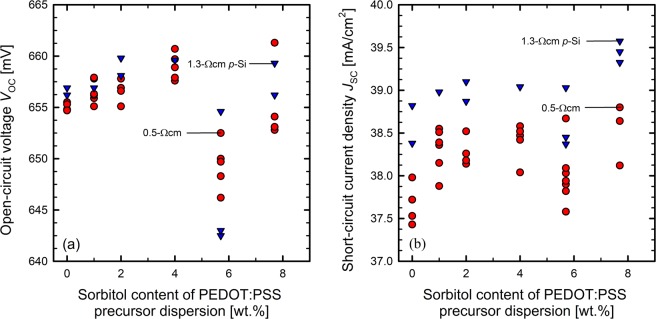
(**a**) Measured *V*_oc_ values of all processed PEDOT:PSS/c-Si solar cells for different amounts of sorbitol in the PEDOT:PSS precursor dispersion. (**b**) Measured *J*_sc_ values of solar cells for different amounts of sorbitol in the PEDOT:PSS precursor dispersion. Solar cells with a base resistivity of 0.5 Ωcm (red circles) and 1.3 Ωcm (blue triangles) are shown.

As the open-circuit voltage *V*_oc_ is very sensitive to recombination losses, the positive impact of the sorbitol admixture observed on the lifetime test samples shown in Fig. 2, should translate to increased *V*_oc_ values on the solar cells with sorbitol admixture. Figure 3(a) shows the measured *V*_oc_ values of each processed BackPEDOT solar cell as a function of the sorbitol concentration in the PEDOT:PSS precursor dispersion. The median parameters of the solar cells with 0.5 Ωcm base resistivity are shown in Table 2 and the parameters of the 1.3 Ωcm cells in Table 3.

**Table 2:** Median and maximum values of the cell parameters for BackPEDOT solar cells with a base resistivity of 0.5 Ωcm.

Sorbitol content in the PEDOT:PSS precursor dispersion [wt.%]	*V*_oc_ [mV]		*J*_sc_ [mA/cm^2^]		*FF* [%]		η [%]		*R*_s_ [Ωcm^2^]	
	Median	Max.	Median	Max.	Median	Max.	Median	Max.	Median	Min.
0.0 (4 cells)	655	656	37.6	38.0	77.6	79.4	19.1	19.8	1.14	0.67
1.0 (6 cells)	656	658	38.4	38.6	79.6	80.7	20.0	20.1	0.72	0.60
2.0 (4 cells)	657	658	38.2	38.5	77.8	80.6	19.6	20.2	1.02	0.62
4.0 (5 cells)	659	661	38.5	38.6	79.7	80.3	20.1	20.4	0.73	0.66
5.7 (6 cells)	650	653	37.9	38.7	74.9	77.8	18.3	19.2	1.66	1.18
7.7 (4 cells)	654	661	38.7	38.9	74.5	75.3	18.9	19.1	1.88	1.52

**Table 3:** Median and maximum values of the cell parameters for BackPEDOT solar cells with a base resistivity of 1.3 Ωcm.

Sorbitol content in the PEDOT:PSS precursor dispersion [wt.%]	*V*_oc_ [mV]		*J*_sc_ [mA/cm^2^]		*FF* [%]		η [%]		*R*_s_ [Ωcm^2^]	
	Median	Max.	Median	Max.	Median	Max.	Median	Max.	Median	Min.
0.0 (2 cells)	657	657	38.6	38.8	75.1	75.6	19.0	19.3	1.55	1.48
1.0 (1 cells)	657	657	39.0	39.0	78.9	78.9	20.2	20.2	0.86	0.86
2.0 (2 cells)	659	660	39.0	39.1	75.4	75.7	19.4	19.4	1.47	1.42
4.0 (1 cells)	660	660	39.0	39.0	78.6	78.6	20.3	20.3	0.89	0.89
5.7 (3 cells)	643	655	38.5	39.0	72.6	76.5	17.9	19.5	1.98	1.27
7.7 (3 cells)	656	659	39.5	39.5	71.1	74.0	18.5	19.3	2.51	1.92

As can be seen from Fig. 3(a) and Tables 2 and 3, *V*_oc_ increases from a median value of 655 mV to 659 mV for the 0.5-Ωcm *p*-Si base material and from 657 mV to 660 mV for the 1.3-Ωcm base material by adding 4.0 wt.% of sorbitol to the PEDOT:PSS precursor dispersion. At a sorbitol concentration of 5.7 wt.%, the median *V*_oc_ values decrease to 650 mV for the 0.5-Ωcm cells and to 643 mV for the 1.3-Ωcm cells. While the improvement in *V*_oc_ by the admixture of up to 4.0 wt.% of sorbitol is in good agreement with our lifetime study shown in Fig. 2, the reduced *V*_oc_ at 5.7 wt.% admixture of sorbitol is unexpected and somewhat peculiar, but seems to be reproducible, as it was observed in all eight cell batches processed within this work. Increasing the sorbitol content further to 7.7 wt.% increases the median *V*_oc_ approximately back to the state without sorbitol admixture. Nevertheless, the maximum *V*_oc_ value of a single cell of 661 mV was achieved on a 0.5-Ωcm cell with 7.7 wt.% sorbitol admixture. From our experimental results obtained on 42 processed BackPEDOT cells we hence conclude that the admixture of sorbitol can have a clear positive impact on the passivation quality of the PEDPOT:PSS/c-Si junction, although not too much of sorbitol should be added. Figure 3(b) compiles the measured *J*_sc_ values of all processed PEDOT:PSS/c-Si solar cells as a function of the sorbitol concentration (median values listed in Tables 2 and 3). The solar cells with 1.3-Ωcm base material provide higher *J*_sc_ values compared to the solar cells fabricated on 0.5-Ωcm base material, which can be attributed to the much higher bulk lifetime in the silicon material with lower doping concentration. Lifetime measurements performed on the 0.5-Ωcm *p*-type FZ-Si material verified that the bulk lifetime of this material is 0.77 ms, whereas that of the 1.3-Ωcm material is 1.56 ms at an excess carrier density Δ*n* of 3 × 10^15^ cm^3^. For both base materials, the addition of sorbitol to the PEDOT:PSS is positively affecting the *J*_sc_ value. For the 0.5-Ωcm material, the median *J*_sc_ increases by 0.7 mA/cm^2^ by adding a sorbitol concentration of 4.0 wt.% to the PEDOT:PSS dispersion. At a higher sorbitol contents of 7.7 wt.% the median *J*_sc_ increases even more by 0.9 mA/cm^2^ compared to the reference case without sorbitol. For the BackPEDOT cells fabricated on the 1.3-Ωcm *p*-Si base material, the median *J*_sc_ increases by 0.8 mA/cm^2^ for a sorbitol concentration of 7.7 wt.% compared to the reference without sorbitol. In this study, the highest measured *J*_sc_ on a PEDOT:PSS/c-Si solar cell with a base resistivity of 1.3 Ωcm is 39.6 mA/cm^2^ for a sorbitol content of 7.7 wt.% of the PEDOT:PSS precursor dispersion. From these results it becomes obvious that the addition of sorbitol to the PEDOT:PSS dispersion effectively reduces the parasitic absorption losses in PEDOT:PSS/c-Si heterojunction cells.

**Figure 4:**
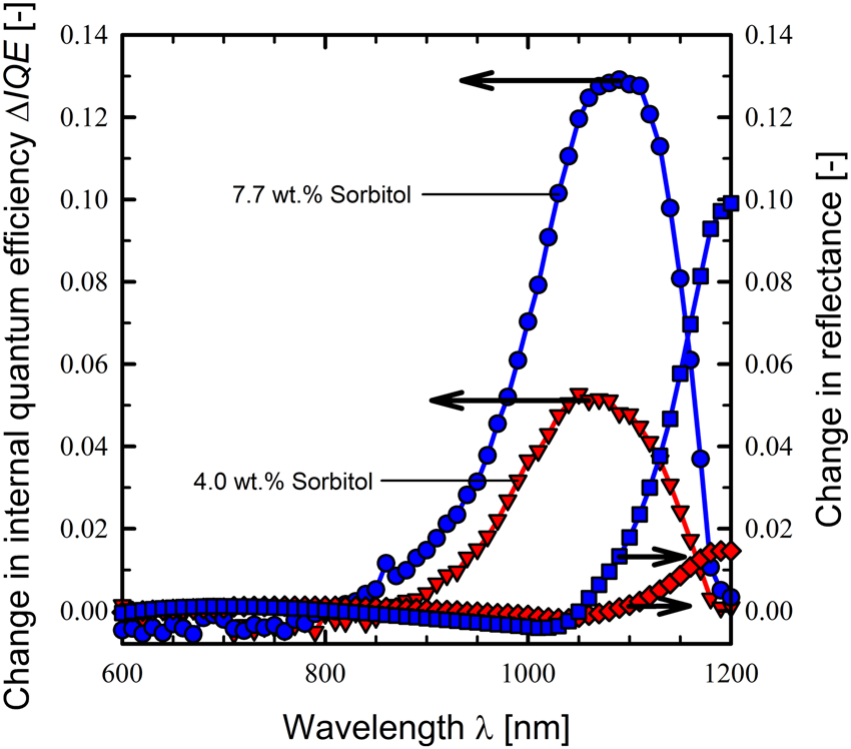
Measured change in the internal quantum efficiency (Δ*IQE*) (blue circles and red triangles) and change in reflectance (blue squares and red diamonds) of exemplary BackPEDOT solar cells by adding 4.0 wt.% (red symbols) and 7.7 wt.% (blue symbols) sorbitol compared to a BackPEDOT solar cell without sorbitol. The solar cells have a *p*-type c-Si base with a resistivity of 0.5 Ωcm and the PEDOT:PSS was spin-coated at 500 rpm for 10 seconds and subsequently at 12000 rpm for 30 seconds.

Figure 4 shows the measured change in the internal quantum efficiencies Δ*IQE* and the change in the reflectance spectra using the LOANA measurement system of two exemplary BackPEDOT solar cells for sorbitol contents in the precursor dispersion of 4.0 and 7.7 wt.%, respectively in comparison to a solar cell without sorbitol in the precursor dispersion. The long-wavelength reflectance above 1000 nm is clearly increased by adding the sorbitol to the PEDOT:PSS precursor dispersion in comparison to the solar cell without admixture of sorbitol, which is attributed to the reduced parasitic absorption in the PEDOT:PSS layer^13^. Moreover, Δ*IQE* is improved in the long-wavelengths range above 800 nm for the solar cells with sorbitol addition, which is attributed to the improvement in the rear passivation of the BackPEDOT cell and in the improved light trapping due to the reduced parasitic absorption within the PEDOT:PSS layer.

**Figure 5:**
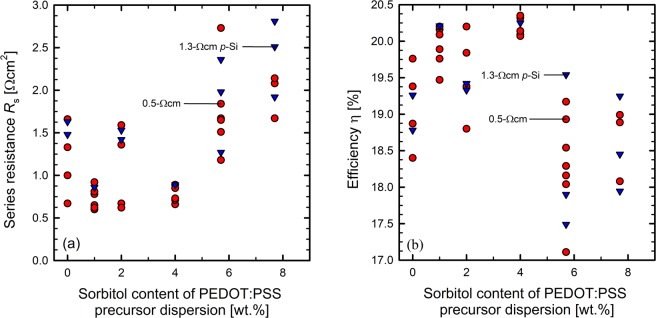
(**a**) Measured series resistance *R*_s_ of the fabricated BackPEDOT solar cells for different amounts of sorbitol in the PEDOT:PSS precursor dispersion. (**b**) Measured solar cell efficiency η of the fabricated solar cells for different amounts of sorbitol in the PEDOT:PSS precursor dispersion. Solar cells have been fabricated on *p*-type silicon wafers with a base resistivity of 0.5 Ωcm (red circles) and 1.3 Ωcm (blue triangles), respectively.

**Figure 6:**
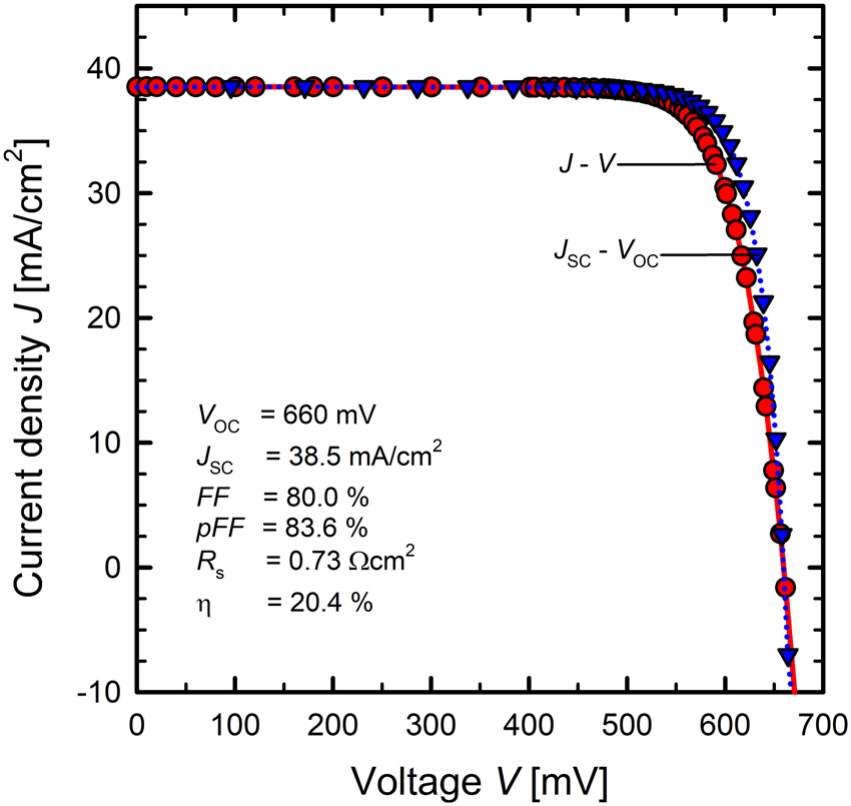
Illuminated *J–V* curve (red circles) and *J*_sc_ − *V*_oc_ curve (blue triangles) of the best solar cell of this study with 4.0 wt.% admixture of sorbitol to the PEDOT:PSS precursor dispersion. The corresponding solar cell parameters are also shown.

Figure 5(a) (and Tables 2 and 3) shows the series resistance *R*_s_ of the fabricated BackPEDOT solar cells as a function of the sorbitol content in the PEDOT:PSS precursor dispersion. Up to a sorbitol concentration of 4.0 wt.% the best *R*_s_ values remain at a constant low level of approximately 0.6 to 1.0 Ωcm^2^. However, there are also outliers with increased series resistance with values around 1.5 Ωcm^2^. At higher sorbitol contents, *R*_s_ shows a pronounced increase with increasing sorbitol content. Median *R*_s_ values are 1.59 Ωcm^2^ for a sorbitol content of 5.7 wt.% and 1.85 Ωcm^2^ for a sorbitol content of 7.7 wt.%. We attribute the increase in the *R*_s_ values for sorbitol contents greater than 4.0 wt.% to a degradation of the solar cell back contact due to the high thermal energy of the electron beam evaporation in combination with a presumably remaining sorbitol content in the PEDOT:PSS layer. Optical microscope images of the metallized cell rear for the different sorbitol concentrations are shown in Supplementary Fig. S1. The amount of sorbitol remaining in the PEDOT:PSS layer is probably higher for sorbitol contents greater 4 wt.% than for lower sorbitol concentrations. Therefore, we observe an increase in *R*_s_ only at high sorbitol concentrations. The best *R*_s_ value realized on a solar cell processed in this study is 0.6 Ωcm^2^ for a sorbitol concentration of 4.0 wt.%, which led to an efficiency of 20.2%. The achieved efficiencies of all fabricated BackPEDOT solar cells as a function of the sorbitol concentration are shown in Fig. 5(b). For PEDOT:PSS/c-Si solar cells manufactured with the PEDOT:PSS dispersion without addition of sorbitol, efficiencies range from 18.4 to 19.7%. By adding a sorbitol content to the precursor dispersion of up to 4.0 wt.%, higher cell efficiencies of up to 20.4% are achieved. If a sorbitol content of more than 4.0 wt.% is added to the precursor dispersion, the cell efficiencies decrease and range from 17.9% to 19.2% at a sorbitol content of 7.7 wt.%. The decrease in solar cell efficiencies with higher sorbitol content is attributed to the increasing *R*_s_ value. The best efficiency measured in this study on a PEDOT:PSS/c-Si solar cell is 20.4% with a sorbitol content in the precursor dispersion of 4.0 wt.%. The illuminated *J*–*V* curve and the *J*_sc_ − *V*_oc_ curve of the best solar cell of this study with the corresponding solar cell parameters is shown in Fig. 6.

The best BackPEDOT solar cell fabricated in this study, shown in Fig. 6, provides a *V*_oc_ of 660 mV and a *J*_sc_ value of 38.5 mA/cm^2^. A good fill factor of 80.0% is achieved due to a low *R*_s_ value of 0.73 Ωcm^2^. This combination of cell parameters leads to the highest efficiency achieved in this study of 20.4% for a PEDOT:PSS/c-Si solar cell with admixture of 4.0 wt.% sorbitol to the precursor dispersion. The *J*_sc_ value of 38.5 mA/cm^2^ achieved on this cell seems relatively low and higher *J*_sc_ values (up to 39.6 mA/cm^2^) were achieved on the 1.3 Ωcm *p*-Si base material, featuring a much higher bulk lifetime compared to the 0.5 Ωcm bulk material. Hence, there seems to be a strong contribution of the bulk recombination in the record cell of our current study and the use of better bulk material should allow to further improve the efficiency.

## Conclusions

In this study, we have fabricated 42 solar cells with phosphorus-diffused front and PEDOT:PSS/c-Si heterojunction at the rear (‘BackPEDOT’ cells). We have examined the impact of adding sorbitol to the PEDOT:PSS precursor dispersion on the solar cell parameters. The recombination current density parameter *J*_0.PEDOT_ of the PEDOT:PSS/c-Si interface showed a median *J*_0.PEDOT_ value of 86 fA/cm^2^ for silicon surfaces passivated with PEDOT:PSS without admixture of sorbitol. At a sorbitol concentration of 7.7 wt.%, the median *J*_0:PEDOT_ value was lowered to only 38 fA/cm^2^ which means that the recombination at the PEDOT:PSS/c-Si interface is effectively suppressed by the addtion of sorbitol. We also observed that the admixture of sorbitol improved the *J*_sc_ values due to the reduced parasitic absorption within the PEDOT:PSS layer, by 0.9 mA/cm^2^ at a sorbitol concentration of 7.7 wt.% on solar cells with a *p*-Si base resistivity of 0.5 Ωcm. The highest achieved *J*_sc_ value was 39.6 mA/cm^2^ on a solar cell with a *p*-Si base resistivity of 1.3 Ωcm and a sorbitol concentration of 7.7 wt.%. Due to the improvement in the surface passivation quality due to the sorbitol admixture, we observed an increase in *V*_oc_ of 4.0 mV on solar cells with a base resistivity of 0.5 Ωcm. An increase of 3.0 mV was observed for solar cells with 1.3 Ωcm base resistivity for admixture of sorbitol contents of 4.0 wt.% to the PEDOT:PSS dispersion. The maximum *V*_oc_ value of a single cell of 661 mV was achieved on a 0.5-Ωcm cell with 7.7 wt.% sorbitol admixture. The series resistance *R*_s_ did not change by up to 4.0 wt.% of sorbitol admixture, but showed a pronounced increase at higher sorbitol concentrations. The optimal sorbitol content concerning efficiency is hence 4.0 wt.%, leading to a median efficiency of 20.2% (5 cells), which is 1.1% absolute higher compared to the median efficiency of the reference cells without sorbitol. The highest efficiency achieved in this study was 20.4% for a sorbitol content of 4.0 wt.%. Our experimental results hence suggest that the admixture of sorbitol increases the efficiency potential PEDOT:PSS/c-Si heterojunction solar cells.

## Supplementary information

Optical microscope images

## Data Availability

All data generated or analyzed during this study are included in this published article.
