# Supplementary material for: Improved surface passivation and reduced parasitic absorption in PEDOT:PSS/c-Si heterojunction solar cells through the admixture of sorbitol
Source: Sci Rep. 2019 Jul 5;9:9775. doi: 10.1038/s41598-019-46280-y (PMC6611827; doi:10.1038/s41598-019-46280-y)
Supplement: Supplementary file 1 — Optical microscope images [file 41598_2019_46280_MOESM1_ESM.docx]

**Improved surface passivation and reduced parasitic absorption in PEDOT:PSS/c-Si heterojunction solar cells through the admixture of sorbitol**

**Marc-Uwe Halbich**^1,3,*^(ORCID: 0000-0003-3341-6785)**, Dimitri Zielke**^1^**, Ralf Gogolin**^1^**, Rüdiger Sauer-Stieglitz**^2^**, Wilfried Lövenich**^2^**, and Jan Schmidt**^1,3^(ORCID: 0000-0002-4851-4452)

^1^Institute for Solar Energy Research Hamelin (ISFH), Am Ohrberg 1, 31860 Emmerthal, Germany

^2^Heraeus Deutschland GmbH&CoKG, Electronic Chemicals, Chempark Leverkusen, 51368 Leverkusen

^3^Department of Solar Energy, Institute of Solid-State Physics, Leibniz University Hannover, Appelstr. 2, 30167 Hannover, Germany

^*^halbich@isfh.de

**Supplementary Information**

| 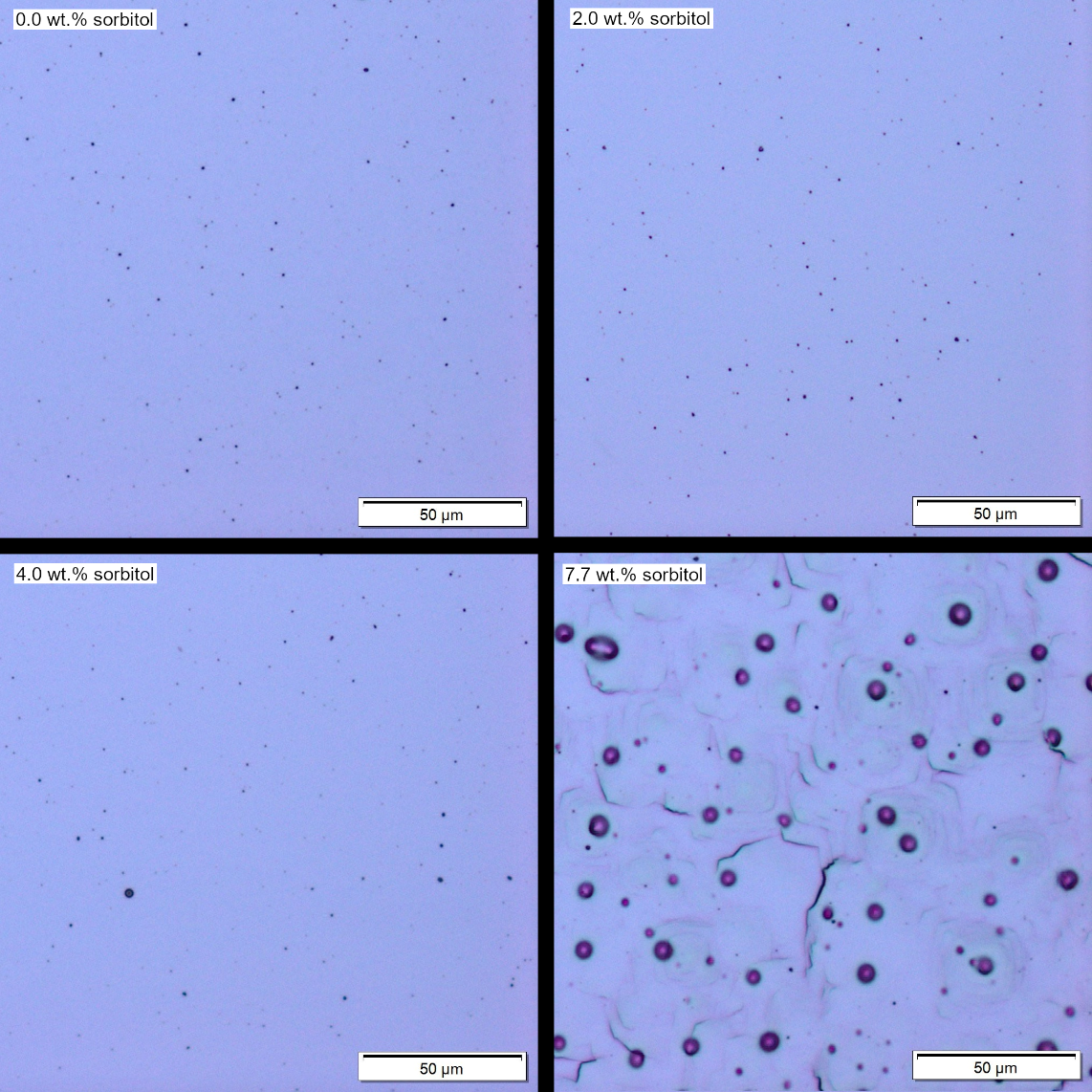 |
| --- |
|  |

**FIGURE S1.** Optical microscope images of the metallized cell rear for different sorbitol concentrations of the PEDOT:PSS precursor dispersion.
